# miR-23a promotes invasion of glioblastoma *via* HOXD10-regulated glial-mesenchymal transition

**DOI:** 10.1038/s41392-018-0033-6

**Published:** 2018-12-28

**Authors:** Kazuhiro Yachi, Masumi Tsuda, Shinji Kohsaka, Lei Wang, Yoshitaka Oda, Satoshi Tanikawa, Yusuke Ohba, Shinya Tanaka

**Affiliations:** 1 Department of Cancer Pathology, Faculty of Medicine, Hokkaido University, Sapporo, Japan; 2 Global Station for Soft Matter, Global Institution for Collaborative Research and Education, Hokkaido University, Sapporo, Japan; 3 Institute for Chemical Reaction Design and Discovery (WPI-ICReDD), Hokkaido University, Sapporo, Japan; 4 Department of Cell Physiology, Faculty of Medicine, Hokkaido University, Sapporo, Japan; 5 Present Address: Division of Cellular Signaling, National Cancer Center Research Institute, Tokyo, Japan

## Abstract

Glioblastoma is the most aggressive and invasive brain tumor and has a poor prognosis; elucidating the underlying molecular mechanisms is essential to select molecular targeted therapies. Here, we investigated the effect of microRNAs on the marked invasiveness of glioblastoma. U373 glioblastoma cells were infected with 140 different microRNAs from an OncomiR library, and the effects of the invasion-related microRNAs and targeted molecules were investigated after repeated Matrigel invasion assays. Screening of the OncomiR library identified miR-23a as a key regulator of glioblastoma invasion. In six glioblastoma cell lines, a positive correlation was detected between the expression levels of miR-23a and invasiveness. A luciferase reporter assay demonstrated that homeobox D10 (HOXD10) was a miR-23a-target molecule, which was verified by high scores from both the PicTar and miRanda algorithms. Forced expression of miR-23a induced expression of invasion-related molecules, including *uPAR*, *RhoA*, and *RhoC*, and altered expression of glial-mesenchymal transition markers such as *Snail*, *Slug*, *MMP2*, *MMP9*, *MMP14*, and *E-cadherin*; however, these changes in expression levels were reversed by HOXD10 overexpression. Thus, miR-23a significantly promoted invasion of glioblastoma cells with polarized formation of focal adhesions, while exogenous HOXD10 overexpression reversed these phenomena. Here, we identify miR-23a-regulated HOXD10 as a pivotal regulator of invasion in glioblastoma, providing a novel mechanism for the aggressive invasiveness of this tumor and providing insight into potential therapeutic targets.

## Cancer: microRNA makes brain cancer invasive

Researchers in Japan have identified key genetic players in an aggressive form of brain cancer. Glioblastoma is the most invasive type of brain tumor, with a five-year survival rate of just 7%. To investigate its invasiveness, a team led by Shinya Tanaka of Hokkaido University tested the effect of 140 microRNAs on glioblastoma cells. They found that miR-23a increased the invasiveness of the cells. Further research revealed that miR-23a reduces the level of the regulatory gene HOXD10 by destroying the protein it encodes. This reduction leads to changes in the expression of genes regulated by HOXD10, increasing affected cells’ invasiveness and altering their morphology. The miR-23a/HOXD10 pathway revealed here not only provides insight into the biology of glioblastoma but also offers a potential therapeutic target.

## Introduction

Glioblastoma (GBM) is the most invasive and aggressive primary brain tumor and has a poor prognosis, showing a 5-year survival rate of 7%. Conventional therapy for GBM involves surgical resection followed by fractionated radiotherapy and concomitant adjuvant chemotherapy with alkylating drugs such as temozolomide (TMZ).^1^ However, the effects of treatment are limited due to the complexity of GBM, involving tumor heterogeneity, rapid invasion, clonal populations maintaining glioma stem cells (GSCs), and a high frequency of recurrence. Genome-wide analyses such as the Cancer Genome Atlas (TCGA) along with other efforts, have identified gene mutations, amplification, modification, and rearrangement as the principal genetic causes of GBM.^2,3^ To date, more than 140 gene mutations have been reported in GBM, most frequently in *EGFR*, *TP53*, *PTEN*, *PIK3CA*, *PIK3R1*, *PDGFRA*, *ATRX*, *IDH1*, *RB1*, *LZTR1*, and *PTPN11*, while TMZ-dependent hypermutations are highly expressed in recurrent tumors.^4–6^ Although various molecular targeted agents have been attempted to be used either as a single agent or in combination therapy, few have been reported to be effective in phase II trials thus far. Notably, many gene mutations in primary tumors are distinct from those in recurrent tumors. In addition, mutations in genes at diagnosis, such as those in *EGFR*, *PDGFRA*, and *TP53*, can switch to different mutations in the same gene at relapse,^5,7,8^ suggesting that complicated spatiotemporal clonal evolution is a primary mechanism of treatment failure. Therefore, new approaches are urgently needed to understand the unique biology of GBM and design optimized therapies.

One unique characteristic of GBM cells is aggressive infiltration and invasion into the surrounding normal tissues along the vascular tracks, preventing complete resection of all malignant cells and limiting the effect of localized radiotherapy. CD44 ligation with hyaluronic acid (HA) has been shown to trigger PI3K/Rho GTPase signaling, leading to GBM invasion *via* regulation of actin polymerization and formation of focal adhesions.^9^ Accumulating evidence has indicated that cancer stem cells (CSCs),^10^ epithelial–mesenchymal transition (EMT) modulated by PI3K/AKT/mTOR signaling,^11^ proneural-mesenchymal shifts *via* NF-κB and JAK-STAT pathways,^12^ angiogenesis-invasion shifts, tumor-derived exosomes^13^ and miRNAs play pivotal roles in GBM migration and invasion. We have previously identified Snail as the master regulator of the irradiation-induced glial-mesenchymal transition (GMT), resulting in promoted migration and invasion.^14^ Thus, a better understanding of the invasive biology of GBM cells is needed to develop innovative therapies to suppress GBM invasion.

MicroRNAs (miRNAs) are small, non coding RNAs ranging from 18 to 24 nucleotides in length that negatively regulate gene expression at the post transcriptional level, primarily through base pairing to the 3′UTR of target mRNA.^15^ Because miRNAs modulate fundamental cell functions such as proliferation, migration, metabolism, and apoptosis,^16^ dysregulation of miRNA expression causes diverse diseases, including cancers.^17,18^ miRNAs can function as tumor suppressor genes or oncogenes and as potential specific cancer biomarkers.^19–21^ Accumulating studies have demonstrated the roles of miRNAs in cancer stem cell self-renewal,^22^ sensitivity to tyrosine kinase inhibitors,^23^ and cancer therapy targeted to the tumor microenvironment.^24^ Several miRNAs have been reported to contribute to the promotion of tumor invasion and metastasis in various cancers, including miR-10b, miR-373, and miR-520c for breast cancer;^25^ miR-17 and miR-19 for colon cancer;^26^ and miR-216a for pancreatic cancer. Recently, the significant role of miRNAs in the pathogenesis of GBM has been increasingly elucidated. In GBM, overexpression of miR-221, miR-10b, miR-130a, miR-125b, miR-9-2, and miR-21 has been reported.^27^ Among these miRNAs, miR-10b, which regulates homeobox D10 (HOXD10), and miR-21, which targets RECK, are important in facilitating glioblastoma invasion.^28,29^

miR-23a has been reported to regulate several physiological phenomena by targeting *MURF1*, *MAFbx*, and *GLS*, leading to promotion of cardiac hypertrophy, inhibition of muscular atrophy, and suppression of glutamine metabolism, respectively.^30^ In addition, dysregulated expression of miR-23a has been reported in various types of human cancers, including upregulation in hepatocellular carcinoma, glioblastoma, bladder cancer, and pancreatic cancer and downregulation in acute promyelocytic leukemia and oral squamous carcinoma.^30^ When overexpressed in cancers, miR-23a directly regulates some target genes such as *NOXA*, *MTSS1*, and *PTEN*, consequently preventing apoptosis, inducing the EMT, and promoting tumorigenesis, respectively.^31^ Recently, miR-23a was shown to be encapsulated in exosomes derived from patients with colorectal cancer,^32^ raising the possibility of its use as a diagnostic and predictive marker. However, the pathobiological role of miR-23a in GBM has remained obscure.

In this study, we identified miR-23a as an oncogene that confers aggressive invasion of GBM cells by directly inhibiting HOXD10 expression. In miR-23a-overexpressing GBM cells, HOXD10 protein levels were dramatically decreased, and mRNA levels of invasion- and GMT-related molecules were markedly altered with polarized formation of focal adhesions, resulting in profound tumor invasion. These findings suggest that miR-23a and HOXD10 are potentially powerful therapeutic targets for GBM treatment.

## Materials and methods

### Cell culture

The human GBM cell lines LN308, LN443, and U373 were kindly provided by Dr. Erwin G. Van Meir (Emory University School of Medicine, Atlanta, Georgia). The KMG4 cell line was kindly provided by Dr. Kazuo Tabuchi (Saga University, Saga, Japan). U87 cells (ATCC#HTB-14) and U251 cells (ATCC#CRL2219) were purchased from the American Type Culture Collection (ATCC). All cell lines, including embryonic kidney 293FT cells, were cultured in Dulbecco’s modified Eagle’s medium (DMEM) (Wako, Osaka, Japan) supplemented with 10% fetal bovine serum (FBS; Invitrogen, Carlsbad, CA, USA) and maintained in a humidified atmosphere of 5% CO_2_ at 37 °C.

### Establishment of miR-23a and HOXD10-overexpressing cells

miR-23a-overexpressing cells were established using the BLOCK-iT HiPerform Lentiviral Pol II miR RNAi Expression System with EmGFP (Invitrogen). 293FT cells were transfected with pLenti6.4/Promoter/MSGW/miR-23a and pLenti6.4/Promoter/MSGW/HOXD10 using Fugene HD transfection reagent (Promega, Madison, WI, USA). After 48 h of incubation, the supernatant was treated with U373 and LN443 glioblastoma cells, and miR-23a and HOXD10-overexpressing cells were selected in DMEM containing blasticidin.

### Anti-miR23a oligonucleotide transfection in KMG4 cells

Anti-miR-23a or comparable scramble oligonucleotides were transfected into KMG4 cells using HiPerfect reagent (Qiagen, Valencia, CA). The sequences utilized were as follows: anti-miR-23a: 5′-AATCCCTGGCAATGTGAT-3′, scramble: 5′-GTGTAACACGTCTATACGCCCA-3′. After 48 h, the cells were utilized for real-time PCR of *Snail*, *MMP2*, *MMP9*, and *MMP12* and for Matrigel invasion assays, as described below.

### Identification of microRNA that promotes glioblastoma invasion

The OncoMir Precursor Virus Library (System Bioscience, Mountain View, CA, USA) was infected into U373 cells, and the Matrigel invasion assay (BD Biosciences, MA, USA) was performed in triplicate as described below. RNA was isolated from cells with elevated invasion ability, and semi quantitative RT-PCR using the OncoMir Precursor Library primers (System Bioscience) and sequencing were performed to identify the infected oncomiRs.

### Matrigel invasion assay

A Matrigel invasion assay was performed as described previously^33^ using a BioCoat Matrigel invasion chamber (24-well chambers) with 8-µm pores (BD Biosciences, MA). U373 and LN443 cells with or without enforced miR-23a and HOXD10 were seeded at a density of 5 × 10^4^ cells into the upper chamber with serum-free medium. Medium containing 10% FBS was added to the lower chamber as a chemo attractant. After incubation for 8 or 24 h, the cells were fixed with 3% paraformaldehyde (PFA) for 10 min and stained with 0.2% crystal violet solution. Non invading cells on the upper surface of each filter were removed by scrubbing. The invaded cells were counted in microscopic fields at ×200 magnification. To minimize bias, cells in at least five randomly selected fields per well were counted. The experiments were performed in triplicate independently, and the mean and standard deviation (SD) of the invading cells were analyzed.

### Prediction of miR-23a-targeting molecules

To predict miR-23a-targeting molecules, PicTar (http://pictar.mdc-berlin.de) and miRanda (http://www.micorna.org) algorithms were used.

### Luciferase reporter assay to target the HOXD10-3’UTR

The HOXD10-3′UTR was amplified from BJ/t cells, converted to cDNA, and sequenced. The HOXD10-3′UTR was cloned into the region downstream of the *Firefly* luciferase gene in a pGL3-promoter luciferase reporter vector (Promega), designated pGL3-SV40-HOXD10. The luciferase reporter vector was co transfected with a miR-23a-overexpression vector (pLenti-6.4/miR-23a) or control vector (pLenti-6.4/nega) into U373 and LN443 cells using Fugene HD transfection reagent (Promega). The *Renilla* luciferase plasmid pCX4-Bleo-RL-Luc (Promega) was utilized as a control for transfection efficiency. After 48 h, a dual-luciferase reporter assay (Promega) was performed as described previously.^34^

### RNA extraction and gene expression analysis

Total RNA from U373 and LN443 cells with or without enforced miR-23a and HOXD10 expression was extracted using an RNeasy Mini kit (Qiagen), and cDNA was synthesized using Superscript VILO (Invitrogen). For semi-quantitative RT-PCR, GoTaq Green Master Mix was utilized, and PCR was performed at 23–33 cycles of denaturation for 30 s at 94 °C, annealing for 30 s at 55 °C, and extension for 30 s at 72 °C. qRT-PCR was performed using a StepOne Real-Time PCR System (Applied Biosystems, Foster City, CA) as described previously.^35^ The primer sequences utilized were as follows: miR-23a: forward 5′-TGCTGGGCCGGCTGGGGTTCCTGGGG-3′, reverse 5′-CCTGGGTCGGTTGGAAATCCCTGGC-3′; *HOXD10*: forward 5′-CTCCACTGTCATGCTCCAGCTCAAC-3′, reverse 5′-CTTTCTGCCACTCTTTGCAGTGAGCC-3′; *RhoA*: forward 5′-CAAGGACCAGTTCCCAGAGGTGTATG-3′, reverse 5′-CTTGACTTCTGGGGTCCACTTTTCTGG-3; *RhoC*: forward 5′-GACACAGCAGGGCAGGAAGACTATG-3′, reverse 5′-GTAGCCAAAGGCACTGATCCGGTTC-3′; *uPAR*: forward 5′-GGCTTGAAGATCACCAGCCTTACCG-3′, reverse 5′-CATCCTTTGGACGCCCTTCTTCACC-3′; *Snail*: forward 5′-GCTGCAGGACTCTAATCCAGA-3′, reverse 5′-ATCTCCGGAGGTGGGATG-3′; *Slug*: forward 5′-TGGTTGCTTCAAGGACACAT-3′, reverse 5′-GTTGCAGTGAGGGCAAGGAA-3′; *E-cadherin*: forward 5′-TCCATTTCTTGGTCTATACGCC-3′, reverse 5′-CACCTTCAGCCATCCTGTTT-3′; *MMP2*: forward 5′-ATAACCTGGATGCCGTCGT-3′, reverse 5′-AGGCACCCTTGAAGAAGTAGC-3′; *MMP9*: forward 5′-GAACCAATCTCACCGACAGG-3′, reverse 5′-GCCACCCGAGTGTAACCATA-3′; *MMP14*: forward 5′-CATTGGGTGTTTGATGAGGCGTCC-3′, reverse 5′-CTCAGGGATCCCTTCCCAGACTTTG-3′; and *glyceraldehyde 3-phosphate dehydrogenase* (*GAPDH*): forward 5′-AGCCACATCGCTCAGACAC-3′, reverse 5′-GCCCAATACGACCAAATCC-3′.

The relative expression levels of total RNA in experimental and control samples were normalized to the *GAPDH* mRNA levels.

### Immunoblotting and antibodies

Immunoblot analyses were carried out as described previously.^36^ Briefly, cells were lysed with RIPA buffer containing 1 mM phenylmethylsulfonyl fluoride (Sigma), 1 mM sodium orthovanadate (Na_3_VO_4_) and a complete protease inhibitor cocktail (Roche) for 10 min on ice. The membrane was treated with primary antibodies (Abs) at 4 °C overnight, followed by incubation with secondary antibodies for 2 h. The primary antibodies were purchased as follows: HoxD10 (E-20) was from Santa Cruz Biotechnology (Santa Cruz, CA), phospho-ERK1/2 was from Cell Signaling Technology (Beverly, MA), and α-tubulin was from Sigma Aldrich. The signals were developed using ECL reagents (GE Healthcare, Little Chalfont, UK) and were visualized using an ImageQuant LAS4000 mini system (Fujifilm, Tokyo, Japan).

### Immunofluorescence for focal adhesions

\U373 and LN443 cells with or without forced miR-23a and HOXD10 expression were cultured on glass-based dishes (IWAKI, Tokyo, Japan) coated with type I-collagen and fixed in 3% PFA in PBS for 15 min. The cells were permeabilized with 0.1% Triton X-100 for 4 min and blocked with 1% BSA for 20 min. To detect focal adhesions, the cells were treated with anti-paxillin Ab (BD Transduction Laboratories, USA) overnight at 4°C, followed by incubation with AlexaFluor488-conjugated anti-mouse IgG (Invitrogen) for 1 h at room temperature (RT). F-actin was stained with AlexaFluor594-conjugated phalloidin (Invitrogen) for 30 min at 37°C. Fluorescent images were obtained using a confocal laser scanning microscope (Olympus, Tokyo, Japan).

### Proliferation assay

U373 and LN443 cells with or without forced miR-23a and HOXD10 expression were seeded into 35-mm dishes at a density of 2 × 10^4^ cells per dish. The medium was changed every 48 h, and the numbers of cells were counted using a cell counter 5 days after cell inoculation.

### Survival analysis of glioma patients

The relationship between HOXD10 expression and survival in glioma patients was analyzed using the public database PrognoScan (http://www.abren.net/PrognoScan/).

### Statistical analysis

All data were represented as the means and SD of experiments performed in triplicate and subjected to one-way analysis of variance, followed by comparison with Student’s *t*-tests. *P* values less than 0.05 were considered statistically significant, as described in the Figure legends.

## Results

### Expression levels of miR-23a are correlated with GBM invasion

**Fig. 1:**
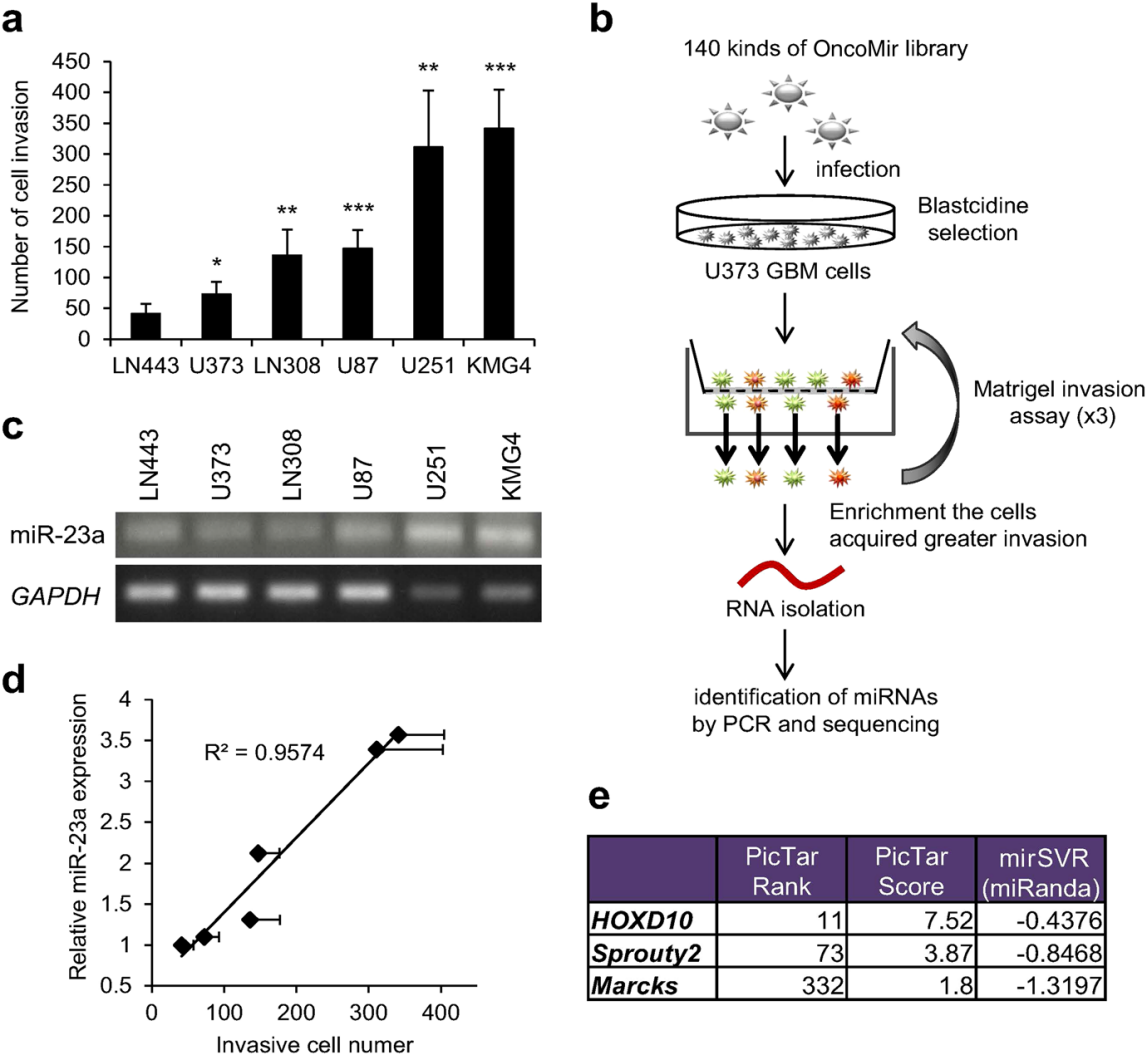
MiR-23a promotes invasion of GBM cells. **a** The Matrigel invasion assays were performed using six human GBM cell lines: LN443, U373, LN308, U87, U251, and KMG4. *P < 0.05, **P < 0.005, and ***P < 0.0005 vs. LN443. **b** Schematic diagram identifying microRNAs to confer marked invasion of GBM cells. U373 cells were infected with the OncoMir Precursor Virus Library, and the Matrigel invasion assay was repeated three times to enrich the cells that acquired elevated invasion ability. Total RNA was isolated from the cells and subjected to semi quantitative RT-PCR using OncoMir Precursor Library primers, followed by sequencing. **c** Endogenous expression levels of miR-23a in the six GBM cell lines were examined by semi quantitative RT-PCR. *GAPDH* was utilized as an internal control. **d** In the six GBM cell lines shown in **c**, the correlations between the invasion ability and expression levels of miR-23a were analyzed. *R*^2^ = 0.95741. **e** The scores for *HOXD10*, *Sprouty2*, and *Marcks* from the PicTar and miRanda algorithms are shown

To evaluate the invasion potential of human GBM, we performed Matrigel invasion assays using six human GBM cell lines. The cells were divided into two groups according to their invasion capabilities: low invasion (LN443, U373, and LN308 cells) and high invasion (U87, U251, and KMG4 cells) (Fig. 1a). To identify microRNAs that confer aggressive invasion in GBM cells, an OncoMir Precursor Virus Library including 140 cancer-related oncomiRs was infected into U373 cells with low intrinsic invasion capabilities. The Matrigel invasion assay was repeated three times to select for the cells that acquired high invasion by oncomiRs (Fig. 1b). Total RNA was isolated, and semi quantitative RT-PCR, followed by sequencing identified miR-23a from the cells that ultimately acquired high invasion (Fig. 1b). miR-23a was expressed in all human GBM cell lines tested with various degrees (Fig. 1c), and the expression levels were significantly correlated with invasion capability (Fig. 1d, *R*^2^ = 0.95741), suggesting the pivotal role of miR-23a in GBM invasion. MiR-181b was also identified in the same context in LN443 cells; however, extrinsic overexpression of miR-181b did not promote invasion in LN443 cells (data not shown).

The PicTar algorithm nominated 472 target genes of miR-23a (data not shown). Among them, three genes, *HOXD10* (11/472), *Sprouty2* (73/472), and *Marcks* (332/472), were implicated in tumor invasion. The distinct algorithm miRanda also identified these genes as miR-23a target genes with low values of mirSVR values (Fig. 1e). The prediction scores from the PicTar and miRanda algorithms for miR-23a targeting *HOXD10* were 7.52 and −0.4376, respectively, nominating *HOXD10* as a reliable target of miR-23a (Fig. 1e).

### MiR-23a directly targets HOXD10 in GBM cells *via* translational regulation

**Fig. 2:**
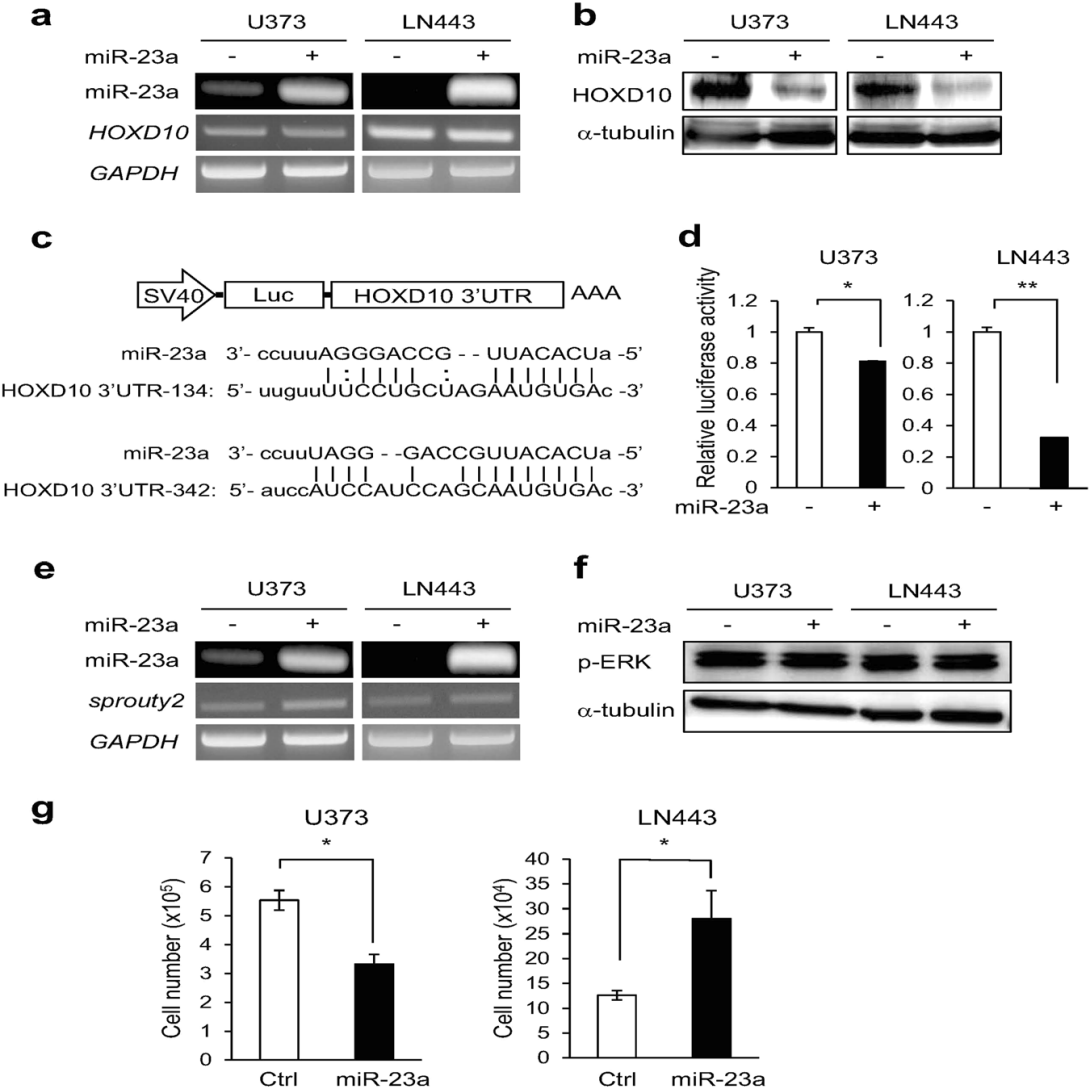
MiR-23a directly targets the HOXD10-3’UTR in GBM cells. **a** U373 and LN443 cells were infected with miR-23a-producing lentivirus or control lentivirus, and the expression levels of miR-23a and *HOXD10* mRNA were examined by semi quantitative RT-PCR. *GAPDH* was utilized as an internal control. **b** The expression levels of HOXD10 protein were examined by immunoblotting in U373 and LN443 cells with or without forced expression of miR-23a. α-Tubulin was used as a loading control. **c** Diagram of the luciferase reporter vector fused to the 3’UTR of *HOXD10* utilized in the luciferase assay. The sequences of miR-23a and the targeted *HOXD10*-3’UTR are shown. **d** Dual luciferase assay. *HOXD10*-3′UTR luciferase activity were measured in miR-23a-overexpressing U373 and LN443 cells. *P < 0.01 and **P < 0.001 vs. without miR-23a. **e** The expression levels of *sprouty2* mRNA in miR-23a-overexpressing U373 and LN443 cells were examined by semi quantitative RT-PCR. **f** The phosphorylation levels of ERK were investigated by immunoblotting in the indicated cells. **g** The cell proliferation of U373 and LN443 cells with or without forced *miR-23a* was investigated and graphed as the means ± SD. * *P* *<* 0.05 vs. control cells

To determine the miR-23a target genes involved in GBM invasion, we established stably miR-23a-overexpressing U373 and LN443 cells by infecting them with miR-23a-producing lentivirus. Although the expression levels of *HOXD10* mRNA were invariant irrespective of miR-23a overexpression (Fig. 2a), the protein levels were significantly decreased by forced miR-23a expression in both cell lines (Fig. 2b), indicating miR-23a-dependent post transcriptional degradation of HOXD10. To analyze whether miR-23a directly targets the *HOXD10*-3′UTR in GBM cells, we developed a luciferase reporter vector fused to the 3′UTR of *HOXD10* (Fig. 2c). In cells stably overexpressing miR-23a, the luciferase activity of *HOXD10*-3′UTR was reduced compared with that in control cells (Fig. 2d), confirming HOXD10 as a direct target of miR-23a. Lower expression of HOXD10 was associated with a shorter survival rate in glioma patients by Kaplan–Meier analysis using the PrognoScan database (data not shown).

Sprouty2 has been reported to suppress invasion of GBM cells by inhibiting Ras GTPase,^37^ raising the possibility that miR-23a might activate the Ras/ERK signaling pathway *via* translational inhibition of Sprouty2, facilitating GBM invasion. However, forced expression of miR-23a had no effect on the expression of *sprouty2* mRNA or the phosphorylation levels of ERK1/2 (Fig. 2e, f). In addition, miR-23a overexpression exhibited a distinct effect on the proliferation of U373 and LN443 cells (Fig. 2f), suggesting a relatively low possibility of Sprouty2 as a universal target of miR-23a.

### MiR-23a regulates the expression levels of invasion- and glial-mesenchymal transition (GMT)-related genes *via* HOXD10

HOXD10 is a member of the Homeobox (Hox) superfamily and has been shown to suppress invasion of GBM by inhibiting the expression levels of *urokinase-type plasminogen activator receptor* (*uPAR)*, *matrix metalloproteinase (MMP) 14*, and *RhoC*.^38^ Therefore, we next examined the effect of miR-23a on the expression of these genes.

**Fig. 3:**
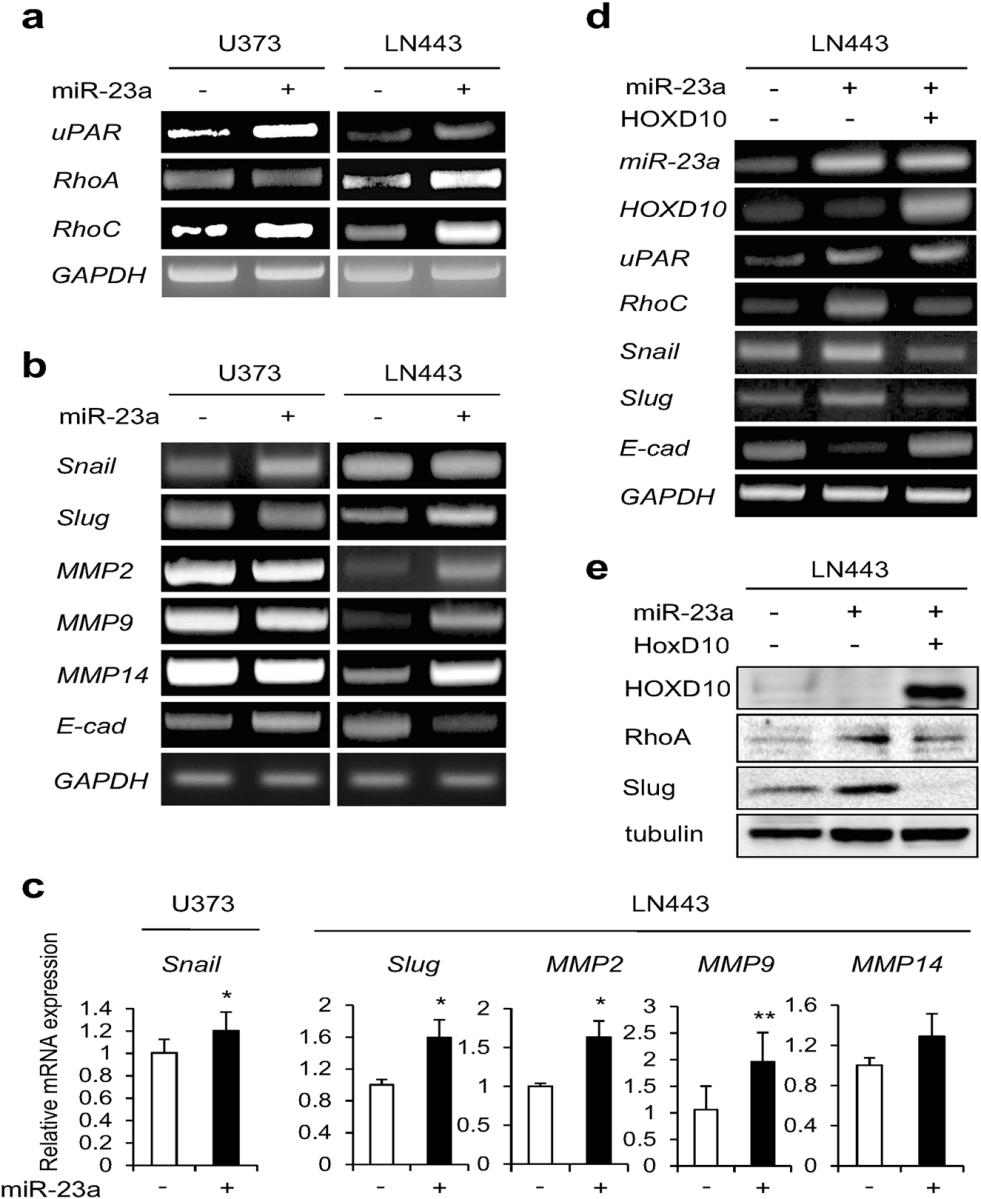
MiR-23a regulates expression of invasion- and glial-mesenchymal transition (GMT)-related genes *via* HOXD10. **a** The expression levels of *uPAR, RhoA, and RhoC* mRNAs were examined in control and miR-23a-overexpressing U373 and LN443 cells by semi quantitative RT-PCR. *GAPDH* was utilized as an internal control. **b**, **c** In U373 and LN443 cells with or without miR-23a overexpression, the mRNA expression levels of the indicated GMT-related genes were investigated by semi quantitative RT-PCR (**b**) and real-time RT-PCR (**c**). *P < 0.05 and **P < 0.005 vs. without miR-23a. **d**, **e** The expression levels of miR-23a and the indicated molecules were examined by semi quantitative RT-PCR (**d**) and immunoblotting (**e**) in LN443 cells with or without miR-23a and HOXD10 overexpression

MiR-23a overexpression markedly increased *uPAR* and *RhoC* expression in both U373 and LN443 cells and *MMP14* and *RhoA* expression in LN443 cells (Fig. 3a, b). We found no miR-23a-dependent elevation in *MMP14* expression in U373 cells, probably due to substantial endogenous expression levels (Fig. 3b). Notably, forced expression of miR-23a triggered marked alterations in the expression levels of glial-mesenchymal transition (GMT)-related genes, as previously reported,^14^ with increased expression of *Snail*, *Slug*, *MMP2*, *MMP9*, and *MMP14* and decreased expression of *E-Cadherin*, especially in LN443 cells (Fig. 3b, c); these genes are perceived as so-called epithelial-mesenchymal transition (EMT)-related genes in other cancers. These alterations were completely reversed by HOXD10 overexpression (Fig. 3d, e), demonstrating the significant contribution of the miR-23a-HOXD10 axis in these gene expression levels.

### MiR-23a produces mesenchymal morphology with polarized focal adhesions

**Fig. 4:**
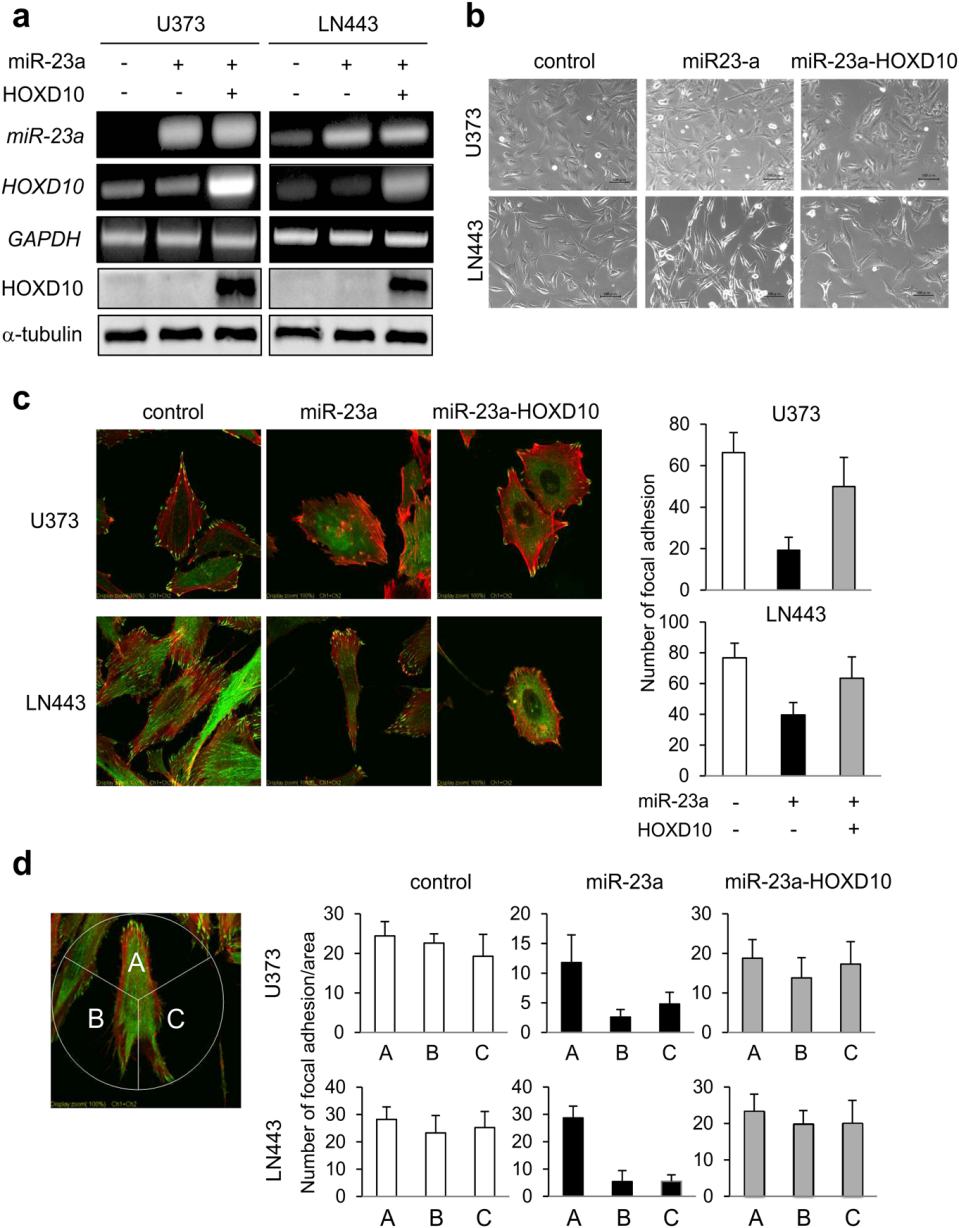
MiR-23a produces mesenchymal changes in cell morphology and affects the polarity of focal adhesions. **a** The expression levels of miR-23a, *HOXD10* mRNA, and HOXD10 protein were examined by semi quantitative RT-PCR (upper three panels) and immunoblotting (lower two panels) in U373 and LN443 cells. *GAPDH* and α-tubulin were utilized as internal controls for semi quantitative RT-PCR and immunoblotting, respectively. **b** Photomicrographs of U373 and LN443 cells with or without miR-23a or HOXD10-overexpression are displayed. The scale bars indicate 100 µm. **c** Immunofluorescence of focal adhesions. (Left panels) U373 and LN443 cells with or without forced miR-23a or HOXD10 expression were subjected to immunofluorescence analysis for paxillin (green) and actin (red). (Right panels) The paxillin counts in the indicated cells are shown. **d** Cells stained with anti-paxillin Ab were divided into three regions by angles of 120°, and the “A” region was set as the movement direction based on cell morphology and the structures of actin filaments. In U373 and LN443 cells with or without forced miR-23a or HOXD10 expression, the paxillin counts were determined and are shown

Because miR-23a significantly alters invasion- and GMT-related gene expression, we further investigated morphological changes with or without miR-23a overexpression (Fig. 4a). Forced expression of miR-23a induced elongation of spindle morphology in U373 and LN443 cells, and HOXD10 overexpression reversed these alterations, returning the morphology to that of control cells (Fig. 4b). Immunofluorescence analysis revealed that the number of focal adhesions represented by paxillin was decreased upon miR-23a overexpression, with shortened actin filaments (Fig. 4c). In addition, extrinsic overexpression of HOXD10 recovered the paxillin count to 80% of that in control cells (Fig. 4c). For a more detailed analysis regarding the assembly of focal adhesions, focal adhesion polarity was investigated with or without miR-23a overexpression. The cell was divided into three regions by angles of 120°, and the “A” region was configured as the movement direction based on cell morphology and the arrangement of actin filaments. In U373 and LN443 cells control cells, paxillin was localized equivalently in all regions (Fig. 4d). However, miR-23a overexpression produced polarity in paxillin distribution, causing significant reductions in the rear regions of cells, namely, the B and C regions (Fig. 4d). Notably, overexpression of HOXD10 abolished the polarity of the focal adhesions (Fig. 4d).

### miR-23a promotes GBM tumor invasion *via* reduced HOXD10

**Fig. 5:**
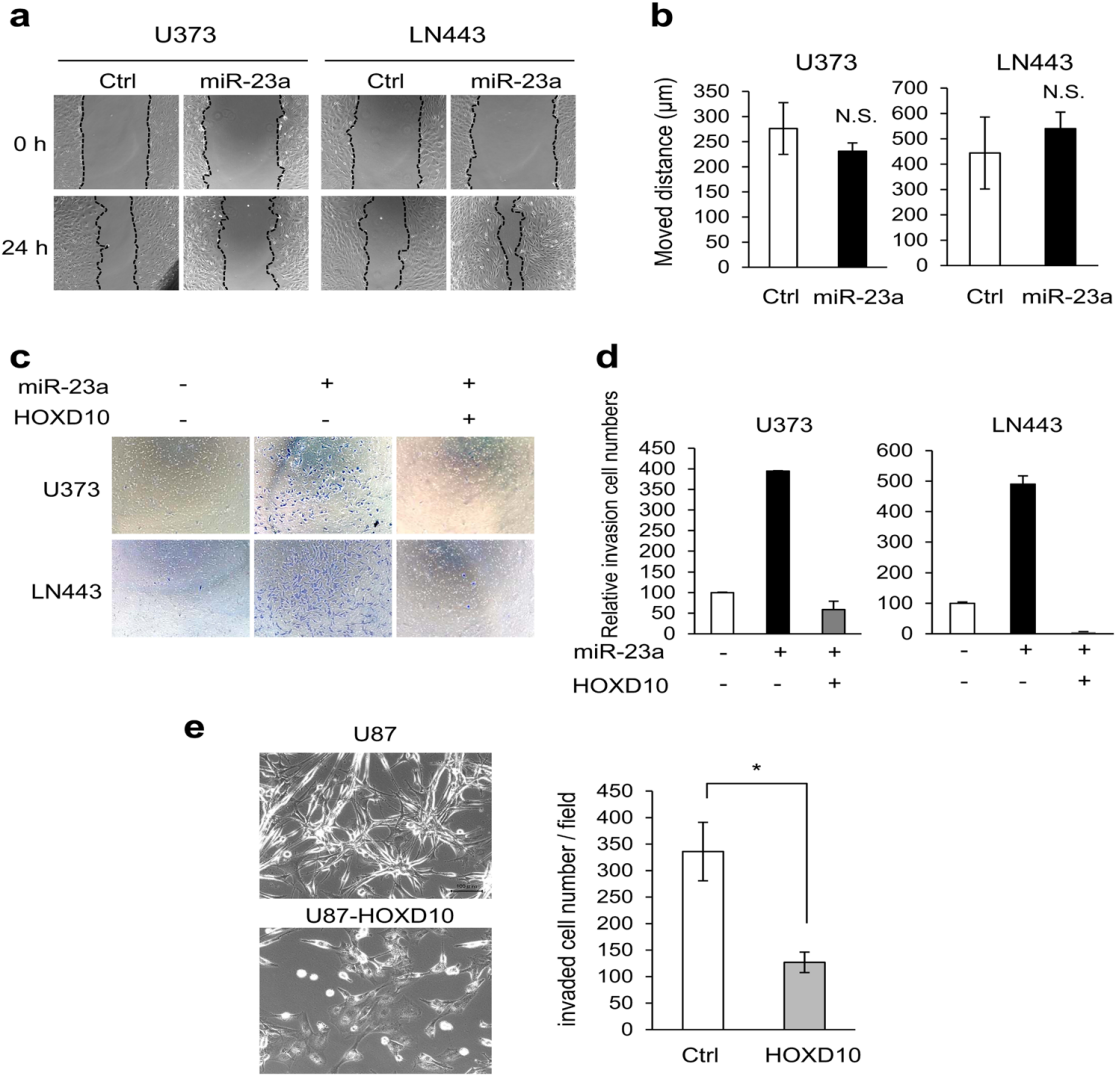
MiR-23a promotes tumor invasion of glioblastoma *via* reduced HOXD10. **a** Wound-healing assays were performed with miR-23a-overexpressing U373 and LN443 cells and their respective control cells. Representative photomicrographs at 0 and 24 h are shown. **b** The distances moved are displayed as the mean ± SD. N.S. indicates not statistically significant. **c** Matrigel invasion assays were performed with both U373 and LN443 cells with or without forced miR-23a or HOXD10 expression. Micrographs of invading cells stained with crystal violet are displayed. **d** In the Matrigel invasion assays, the invaded cells under the filter were counted in three randomly selected regions, and graphed as the mean ± SD. **e** (Left) Micrographs of U87 cells with or without forced HOXD10 expression are displayed. (Right) In the Matrigel invasion assays, invaded cells were counted, and the data are presented as the mean ± SD. * *P* < 0.01 vs. control cells

**Fig. 6:**
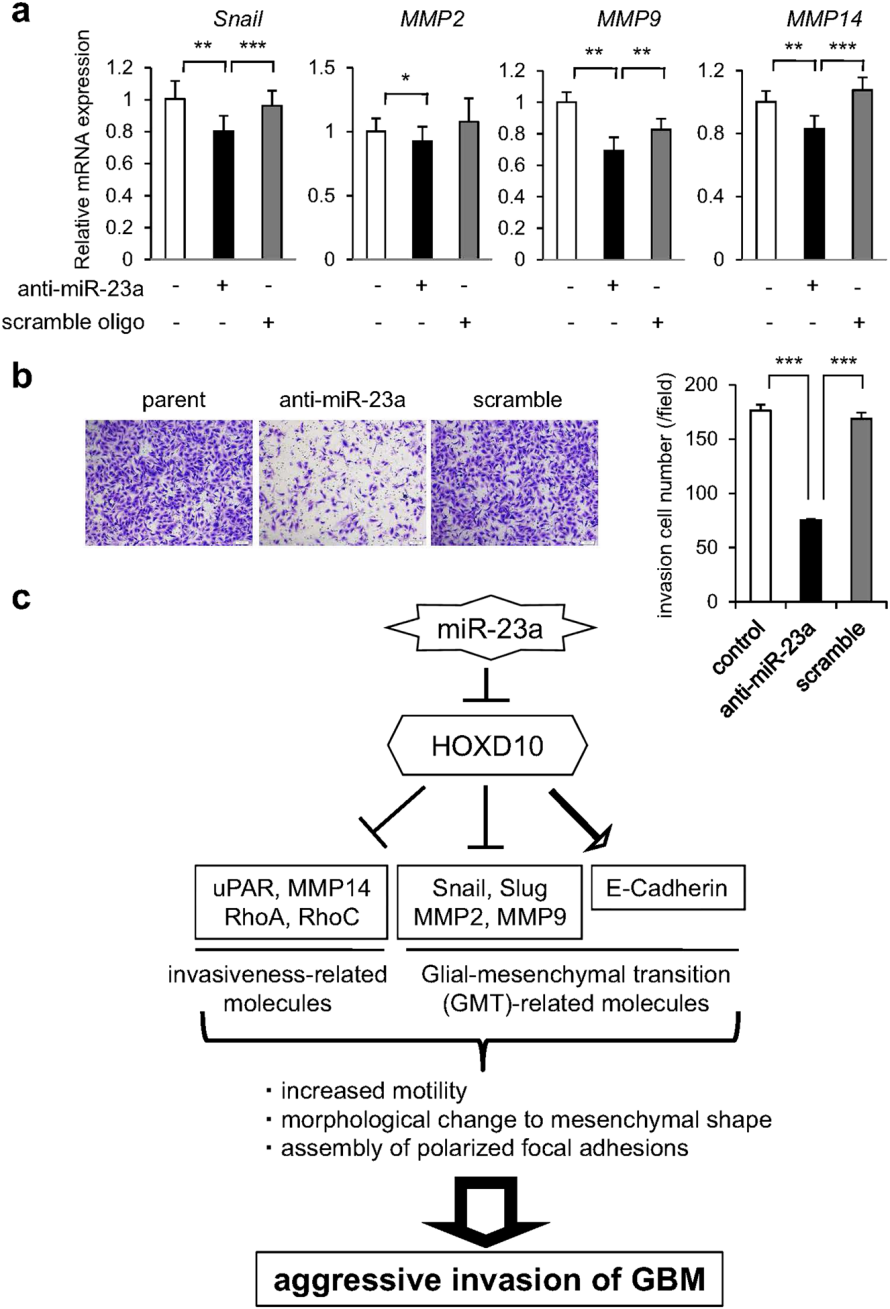
Mechanisms of miR-23a-regulated promotion of GBM invasion through targeting of HOXD10. **a**, **b** KMG4 cells were transfected with anti-miR-23a and scramble DNA as a control, and the expression levels of *Snail*, *MMP2*, *MMP9*, and *MMP14* (**a**) and invasion ability (**b**) were investigated. *P < 0.05, **P < 0.005, and ***P < 0.0005 vs. the indicated samples. **c** miR-23a directly targets the *HOXD10*-3′-UTR, triggering dramatic alterations in the expression of genes associated with invasion (*uPAR*, *MMP14*, *RhoA*, and *RhoC*) and glial-mesenchymal transition (GMT) events (*Snail*, *Slug*, *MMP2*, *MMP9*, and *E-cadherin*), and inducing polarity of focal adhesions, ultimately resulting in cooperatively aggressive invasion of GBM

To assess the effectiveness of miR-23a in GBM cell migration and invasion, we performed a wound-healing assay and a Matrigel invasion assay using U373 and LN443 cells with or without extrinsic miR-23a expression. In the wound-healing assay, the effect of miR-23a on cell motility alone differed across cell types (Fig. 5a, b). However, miR-23a overexpression strikingly promoted invasion in both U373 and LN443 cells to levels 4.0-fold and 5.0-fold higher than those in control cells, respectively (Fig. 5c, d). These increases were reversed by extrinsic expression of HOXD10 (Fig. 5c, d). U87 cells with intrinsic high expression of miR-23a natively possessed higher invasion potential (Fig. 1a, c). Enhanced expression of HOXD10 triggered dramatic alterations in morphologies, changing cells from having a distinct piled-up spindle shape to having a flat shape with an enlarged cytoplasmic compartment and resulting in a substantial decline in invasiveness (Fig. 5c). Anti-miR-23a oligonucleotide treatment to KMG4 cells, which had the highest miR-23a expression and invasiveness, significantly decreased the expression levels of *Snail*, *MMP2*, *MMP9*, and *MMP14* (Fig. 6a), resulting in marked suppression of invasion (Fig. 6b).

## Discussion

GBM is an extremely aggressive tumor with a 5-year survival rate of 7% due to high invasion into surrounding normal brain tissue; elucidation of the underlying molecular mechanisms is therefore essential to develop effective therapies and improve prognoses. In this study, we addressed a part of this long-standing issue using the OncoMir library infection system and found that miR-23a directly targets the *HOXD10*-3′UTR and promotes tumor cell invasion by elevating the expression levels of invasion- and glial-mesenchymal transition (GMT)-related genes and inducing polarity of focal adhesions in GBM (Fig. 6c).

In this analysis, two GBM cell lines, LN443 and U373, with intrinsically low invasion potential were infected with the OncoMir library. After repeating the Matrigel invasion assay, we identified miR-23a as the microRNA responsible for the conferring on U373 cells aggressive invasion capabilities (Fig. 1b). In addition, miR-181b was also identified in the same context in LN443 cells. However, extrinsic overexpression of miR-181b did not promote invasion in LN443 cells (data not shown), in accordance with a previous report showing miR-181b-suppressed invasion in GBM.^39^ Therefore, we focused on the role of miR-23a. cAMP response element-binding protein 1 (CREB1) directly binds to the promoter of miR-23a to promote its expression, while STAT3 indirectly induces miR-23a expression.^40^ Given that both CREB1 and STAT3 are up regulated in glioma,^40,41^ transcription factor-dependent upregulation of miR-23a might contribute to the aggressive invasion of GBM.

*Hox* superfamily genes, including *HOXD10*, encode transcriptional factors regulating cell differentiation and morphogenesis during development.^42^ Dysregulation of the *Hox* gene disrupts various signaling pathways related to tumorigenesis and metastasis.^43^ A positive correlation exists between reductions in *HOXD10* mRNA and increased malignancy of breast cancer and endometrial adenocarcinoma.^44^ Forced expression of *HOXD10* mRNA strikingly suppresses tumor motility and invasion in breast cancer,^45^ suggesting a potent inhibitory role of HOXD10 in tumor invasion. In GBM, miR-10b has been previously reported to inhibit invasiveness by targeting *HOXD10* and regulating the transcription of *MMP14* and *uPAR*.^28^ Previously, miR-23a has been reported to regulate expression of HOXD10 in glioma cells.^46^ In this study, we also identified miR-23a as a novel direct regulator of HOXD10 *via* translational but not transcriptional regulation (Fig. 2a, b). Forced expression of miR-23a increased the expression levels of *uPAR*, *RhoC*, *Snail*, and *Slug* but decreased those of *E-cadherin* in a HOXD10-dependent manner (Fig. 3d, e). *uPAR*, *MMP14*, *RhoC*, and *RhoA* have been reported to be targets of miR-23a.^38^ MMP14 seems to regulate the expression levels of *MMP2* and *MMP9* in inflammatory breast cancer.^47^ Notably, our findings suggest that the miR-23a-HOXD10 axis is a novel regulator of the expression of *Snail*, *Slug*, and *E-cadherin*, which are GMT-regulated genes in GBM. Taken together, the results suggest that miR-23a-triggered consecutive gene expression might evoke aggressive invasion of GBM cells with extensive vascularization through degradation of extracellular matrices.

MiR-23a overexpression induced polarity in the distribution of focal adhesions as shown by paxillin, which was completely reversed by additional forced expression of HOXD10 (Fig. 4c, d). RhoA has been shown to reduce interactions between focal adhesion proteins by partly inhibiting the phosphorylation of paxillin, causing reassembly of the actin cytoskeleton, which leads to invasion and migration of melanoma and breast cancer.^48^ In addition, RhoC induces colocalization of focal adhesion components such as paxillin, paxillin kinase linker (PKL), and FAK, resulting in promoted invasion in prostate cancer cells.^49^ Based on this evidence, spatiotemporal coordination of RhoA and RhoC might produce miR-23a-induced polarity of focal adhesions, one of pivotal events promoting the high invasiveness of GBM.

Although kinase-targeted therapy seems to be a promising therapeutic approach in GBM, such therapies have been ineffective in the clinical setting due to the complexity of GBM with regards to spatiotemporal clonal evolution. Therefore, a kinase-irrelevant strategy using anti-miRNAs might be an innovative and effective approach to target numerous genes. A distinct effect of miR-23a on cell growth was observed in LN443 and U373 cells (Fig. 2g), likely because miR-23a regulates different targets; the PicTar algorithm predicted 472 genes as direct targets of miR-23a. Because the effect of miR-23a on cell growth remains controversial and is dependent on the cellular context, special attention should be paid to assessing the subset of miR-23a-targeted genes that determines the pathophysiological properties of GBM cells.

Our studies demonstrated that glioblastoma cells acquire prominent invasive potential *via* a miR-23a-HOXD10-GMT-related pathway. Forced expression of miR-23a promotes invasion by directly targeting the *HOXD10*-3′UTR. Upregulation of the HOXD10 protein by miR-23a depletion might be an effective approach to suppress invasion of human GBM.
